# Editorial: Patients-oriented treatments for chronic inflammatory skin diseases

**DOI:** 10.3389/fmed.2024.1473753

**Published:** 2024-09-17

**Authors:** Luca Mastorino, Simone Ribero, Martina Burlando, Pedro Mendes-Bastos

**Affiliations:** ^1^Dermatologic Clinic, Department of Medical Sciences, University of Turin, Turin, Italy; ^2^Department of Health Sciences, DISSAL, University of Genoa, Genoa, Italy; ^3^Dermatology Center, Hospital CUF Descobertas, Lisboa, Portugal

**Keywords:** psoriasis, hidradenitis suppurativa (HS)/acne inversa therapy, atopic dermatitis, biologics, bimekizumab, brodalumab, supplement

The therapeutic options for the treatment of inflammatory skin conditions, namely psoriasis disease, atopic dermatitis (AD), and hidradenitis suppurativa (HS), have increased considerably in recent years, following the availability of biologics and small molecules (1–3). These molecules have demonstrated greater efficacy and safety compared to traditional systemic drugs, and have improved treatment targets in both clinical and patient-reported outcomes (1–3).

Many of these drugs have been demonstrated to be effective and safe in the long term (1–3).

On the other hand, the wide range of treatments now available can significantly complicate the clinician's task of selecting the best option for each patient suffering from an inflammatory skin disease (4). Direct comparisons between the more recent treatments are still scarce, involving only a few of the available molecules for AD and psoriasis (4). To date, no guidelines based on phenotypic patient characteristics are available to guide the clinician toward a more specific treatment (4).

To proceed with a tailor-made treatment selection, it is necessary to identify shared treatment targets as already proposed in several consensus recommendations for psoriasis, and more recently for AD. Gisondi et al. have suggested that the achievement of PASI (Psoriasis Area Severity Index) 90 and DLQI (Dermatology Life Quality Index) 0/1 within 16 weeks as an ideal psoriasis treatment target, while guaranteeing safety, reducing progression to psoriatic arthritis (PsA), and ensuring a good treatment retention rate (5).

In this regard, the real-life experience of Gargiulo et al. in a retrospective cohort published in this Research Topic showed a rapid response to bimekizumab (IL-17A/F inhibitor), which was recently approved for the treatment of psoriasis and PsA. More than 97% and 75% of treated patients achieved PASI75 and PASI100, respectively, at 16 weeks, with no substantial differences between bio-naive and experienced patients, and no major adverse events. In another study, the same author presented real-life data of brodalumab (IL-17RA inhibitor) at 3 years. Brodalumab's drug survival was >85% at 3 years, with no identified factors predicting discontinuation, which happened due to adverse events in only 3% of cases. Significant differences in effectiveness were only observed in the bio-experienced group of patients, with minimal impact of joint involvement, difficult to treat sites, and cardiovascular comorbidities on response.

Concerning the impact on quality of life (QoL), Belachew et al., in a large cohort of psoriatic patients attending an Ethiopian Dermatology center, observed that male gender, long duration of treatment and disease, low income, use of alternative therapies, and presence of comorbidities had a significant negative impact on quality of life outcomes.

Lobão et al. discussed the role of CD8^+^ T_RM_ and T_reg_ cells in the pathogenesis and possible progression from psoriasis to PsA, pointing out that guselkumab showed an increase in the ratio of the latter cell population to the former, allowing better long-term results than secukinumab. The authors speculate that inhibiting IL-23 may more effectively reduce the inflammatory conditions that promote the progression of psoriatic disease in joints compared to inhibiting IL-17.

As ideal targets for AD, De Bruin-Weller et al. identified the achievement of EASI ≤ 7 or EASI75, patient-oriented eczema measure (POEM) ≤ 7, SCORAD (Scoring AD) 75 or ≤ 24, DLQI ≤ 5, PP-NRS (peak pruritus numerical rating scale) ≤ 4 within 6 months of treatment, showing a greater impact on patient-reported outcomes in this condition compared to psoriasis (6).

To date, an ideal therapeutic target for HS has not yet been defined, as no available treatments achieve clinically satisfactory responses despite the recent expansion of therapeutic options. No consensual selection of appropriate scores for assessment of this disease has been reached, although different scales such as HiSCR (HS clinical response), IHS4 (HS severity score system), Sartorius, Hurley stage, and HiSqOL (HS quality of life) are available (3). In this Research Topic, Macca et al. highlighted the protean aspect of the disease, not only discussing the possible treatments but also the possible syndromic associations with other inflammatory diseases such as psoriasis, PsA, pyoderma gangrenosum, acne, ankylosing spondylitis, and septic arthritis.

The same authors highlight the unsatisfactory efficacy of the available treatments, noting that antibiotics, TNF-α inhibitors (adalimumab, infliximab, golimumab), and IL-1 inhibitors (canakinumab, anakinra) unfortunately only work in some patients and not in all. However, they emphasized the role of the IL-17 axis in the immunopathogenesis of the disease, which gives us hope for specific inhibitors, particularly secukinumab and bimekizumab. Although there may be a possible role for IL-23 inhibition for the treatment of HS, IL-23 inhibitors have recently shown poor efficacy in pivotal trials.

Defining a treatment target is essential for selecting patient endo-phenotypes that respond better to one treatment compared to others. Another obstacle is the variation in access to treatment options in different countries and even within different regions of the same country.

The Ethiopian study mentioned earlier indirectly highlights the poor access to modern biologic drugs in the study population, as no such treatments administered and the disease was primarily managed with topical therapies; only 4.5% of patients received a systemic drug like methotrexate. Allowing treatment choices based on individual patient characteristics is always preferable to making decisions solely based on cost-effectiveness (4).

Modern treatments can be use even for highly selected patients, as may be the case with rare diseases. Niedźwiedź et al. report the case of an 11-year-old child suffering from CARD14-associated papulosquamous eruption (CAPE), in whom after a therapeutic failure with adalimumab, careful modulation with ustekinumab, administered every 8 weeks, made it possible to achieve a CDLQI (Child-DLQI) of 0 and an FDLQI (Family-DLQI) of 3 in a short time.

Finally, the phase II study by Ehst et al., investigating the gut-skin axis in psoriasis, showed a superior therapeutic response vs. placebo of a new immunomodulatory preparation derived from Prevotella histicola, paving the way for possible oral supplementation for therapeutic or supportive purposes in the management of psoriatic disease.

In conclusion, treatment based on the clinical characteristics of the patient with an inflammatory skin disease appears preferable to selection based solely on national/international guidelines or cost-effectiveness. Modern laboratory techniques, such as immunophenotyping or therapeutic drug monitoring, seem promising and will play an essential role supporting therapeutic decision-making in the future (7, 8). The journey toward personalized treatment involves selecting the appropriate molecular targets and identifying common clinical targets to make a more precise therapeutic decision that also considers the patient's individual needs.
